# Integrated Paleoenvironmental Reconstruction and Taphonomy of a Unique Upper Cretaceous Vertebrate-Bearing Locality (Velaux, Southeastern France)

**DOI:** 10.1371/journal.pone.0134231

**Published:** 2015-08-19

**Authors:** Aude Cincotta, Johan Yans, Pascal Godefroit, Géraldine Garcia, Jean Dejax, Mouloud Benammi, Sauveur Amico, Xavier Valentin

**Affiliations:** 1 Department of Geology, NaGRIDD, University of Namur, Namur, Belgium; 2 Directorate ‘Earth and History of Life’, Royal Belgian Institute of Natural Sciences, Brussels, Belgium; 3 Institut de Paléoprimatologie, Paléontologie Humaine: Evolution et Paléoenvironnements UMR 7262, University of Poitiers TSA 51106, Poitiers, France; 4 Centre de Recherches sur la Paléobiodiversité et les Paléoenvironnements (CR2P, UMR 7207), Sorbonne Universités- MNHN, CNRS, UPMC Paris 6. Muséum national d’Histoire naturelle, Paris, France; 5 Hôtel du Département, Direction de l’Environnement, Conseil Général des Bouches-du-Rhône, Marseille, France; 6 Palaios association, Valdivienne, France; University of Pennsylvania, UNITED STATES

## Abstract

The Velaux-La Bastide Neuve fossil-bearing site (Bouches-du-Rhône, France) has yielded a diverse vertebrate assemblage dominated by dinosaurs, including the titanosaur *Atsinganosaurus velauciensis*. We here provide a complete inventory of vertebrate fossils collected during two large-scale field campaigns. Numerous crocodilian teeth occur together with complete skulls. Pterosaur, hybodont shark and fish elements are also represented but uncommon. Magnetostratigraphic analyses associated with biostratigraphic data from dinosaur eggshell and charophytes suggest a Late Campanian age for the locality. Lithologic and taphonomic studies, associated with microfacies and palynofacies analyses, indicate a fluvial setting of moderate energy with broad floodplain. Palynomorphs are quite rare; only three taxa of pollen grains occur: a bisaccate taxon, a second form probably belonging to the Normapolles complex, and another tricolporate taxon. Despite the good state of preservation, these taxa are generally difficult to identify, since they are scarce and have a very minute size. Most of the vertebrate remains are well preserved and suggest transport of the carcasses over short distances before accumulation in channel and overbank facies, together with reworked Aptian grains of glauconite, followed by a rapid burial. The bones accumulated in three thin layers that differ by their depositional modes and their taphonomic histories. Numerous calcareous and iron oxides-rich paleosols developed on the floodplain, suggesting an alternating dry and humid climate in the region during the Late Campanian.

## Introduction

**Fig 1:**
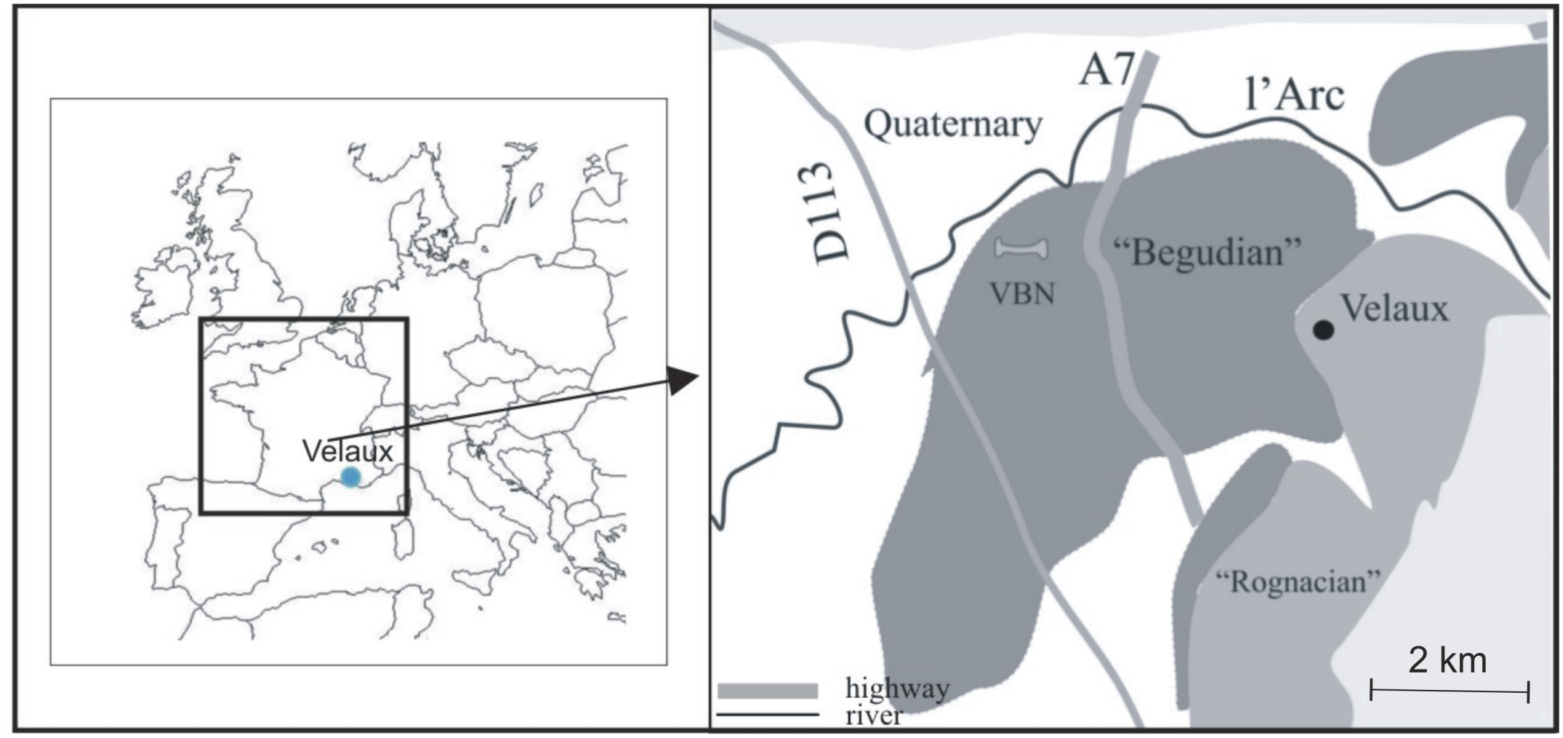
Geographical and Geological maps of Velaux-La Bastide Neuve (VBN) area. VBN is located in southeastern France, between Marseille and Aix-en-Provence. Our fossil-bearing locality is indicated by the bone, between two major roads (D113 and A7). The site belongs to the “Begudian” local stage.

Late Cretaceous continental deposits are widely exposed in southern France and have yielded numerous and diverse vertebrate remains [1] especially in the Aix-en-Provence Basin (e.g. [2], [3], and [4]). However, few studies of both sedimentology (including lithofacies, microfacies, and palynofacies) and vertebrate taphonomy have been conducted at these localities (e.g. [5]). The Velaux-La Bastide Neuve site (Fig 1) was discovered in 1992 by one of us (X.V.) and hundreds of vertebrate remains have been collected during an initial survey (2002) and two large-scale field campaigns (2009 and 2012). The vertebrate assemblage represents a highly diverse fauna including chelonian, crocodilian, dinosaur and pterosaurs. Among the dinosaurs, a new titanosaur genus, *Atsinganosaurus velauciensis*, was described based on partially articulated skeletons [6]. The present paper aims to describe the sedimentological and taphonomic context of the Velaux locality and to reconstruct its paleoenvironment, using lithofacies, microfacies, palynofacies associated with taphonomy and fossil descriptions, and also provide a complete inventory of the vertebrate taxa found in this area.

## Geological Setting and Stratigraphy

The Velaux-La Bastide Neuve fossil-bearing site is located in the western part of the Aix-en-Provence Basin (Bouches-du-Rhône department, southeastern France), an east-west oriented syncline of about 400 km^2^ [7]. The basin fill is composed of fluvio-lacustrine deposits dating from Santonian to Lutetian age [8] that were deposited following an episode of epeirogeny at the end of the Santonian [9]. The age of the site is based on continental biostratigraphic data such as charophytes and dinosaur eggshell. The continental layers exposed in Velaux-La Bastide Neuve site were previously attributed to the “Begudian” local stage (Fig 1), correlated to the Late Campanian [10]. Two charophyte biozones have been recognized at Velaux-La Bastide Neuve. The *Peckichara pectinata* biozone is correlated to the middle-early Late Campanian, and the *Peckichara cancellata* biozone to the Late Campanian [5]. In addition, dinosaur eggshell from this locality belong to the *Megaloolithus aureliensis* biozone, correlated to the Late Campanian [10].

## Material and Methods

During the two field campaigns a surface of 375 m^2^ to a depth of 1.2 m was excavated, which resulted in 100 m^3^ of overburden and matrix. During the first campaign (2009), several small areas separated from the main fossil-bearing layer were worked. In 2012, two large sections were excavated to complete the previous sampling. A total of 308 fossil specimens were inventoried during the two field campaigns- they are housed at the Moulin seigneurial Museum and at the Henri-Ricard Archaeological repository, both located in Velaux.

**Fig 2:**
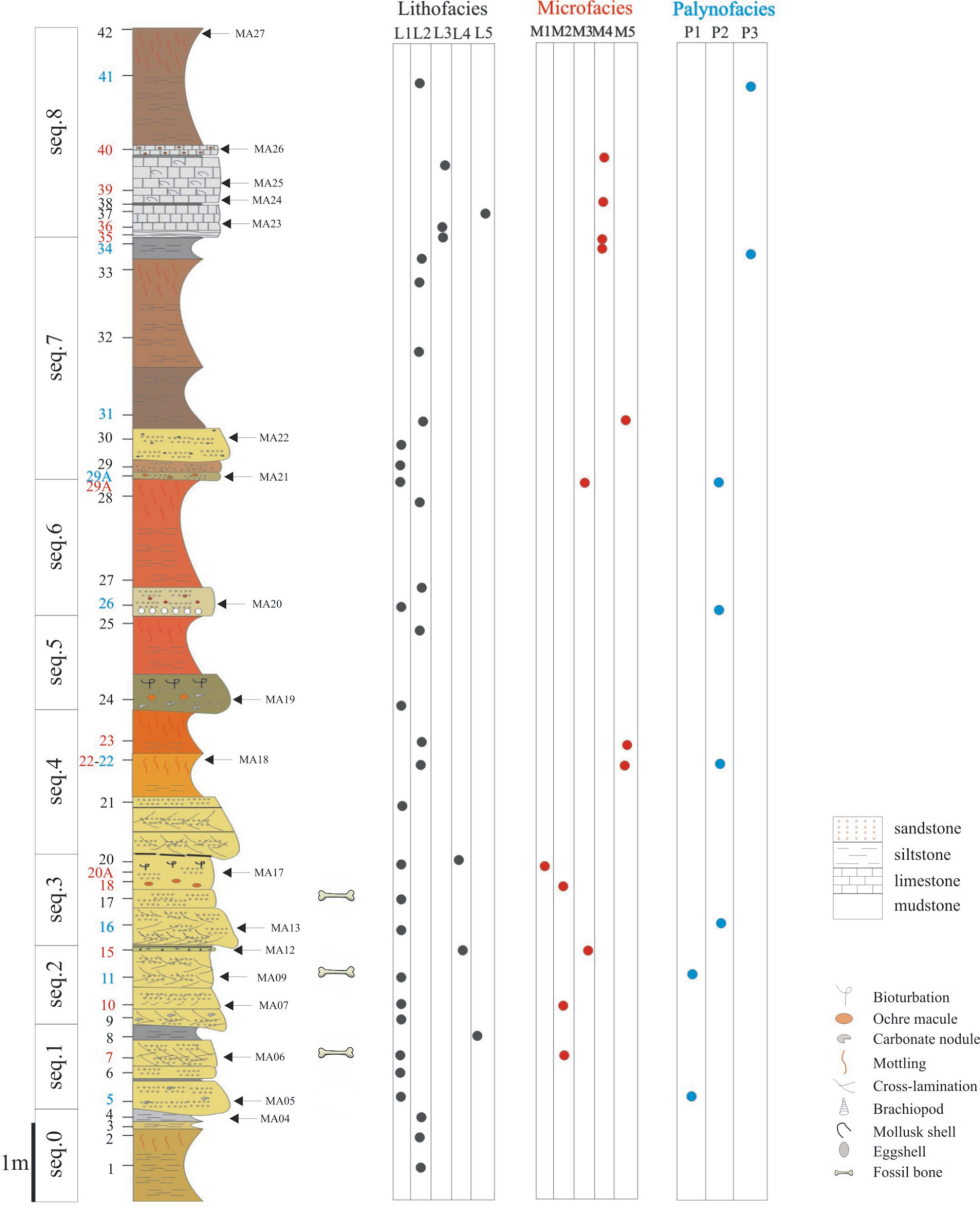
Sedimentological succession of Velaux-La Bastide Neuve locality. Three vertebrate-bearing layers are present: in the sequences 1, 2 and 3. Numbers on the left side correspond to samplings; blue numbers are palynofacies samples; red numbers are microfacies samples and black ones are lithofacies samples. The three tables on the right discriminate the different facies for these samples. Location of magnetostratigraphic samples are indicated by arrows, and the letters MA.

Rock samples for sedimentological analyses were collected from each layer of the sedimentological section (Fig 2). Sampling for several types of analysis was performed at the same time; 18 samples were collected for magnetostratigraphic analyses. Forty one samples were used to study the lithofacies; among these, eight were used for palynological processing, and thirteen were used to prepare thin sections for microfacies analysis. Palynological samples were prepared using standard methods [11]. Two samples (VBN-18 and VBN-20A) collected from sandy horizons were used for isotopic dating using standard K-Ar methods [12].

### Magnetostratigraphy

Oriented block samples were collected from 27 stratigraphic levels in the 16.3 m thick section. The paleomagnetic analyses were carried out at the Laboratory of the iPHEP (University of Poitiers). The intensity and direction of remanent magnetization were measured with a JR6 spinner magnetometer using four position standard specimen holders.

### Taphonomy

**Fig 3:**
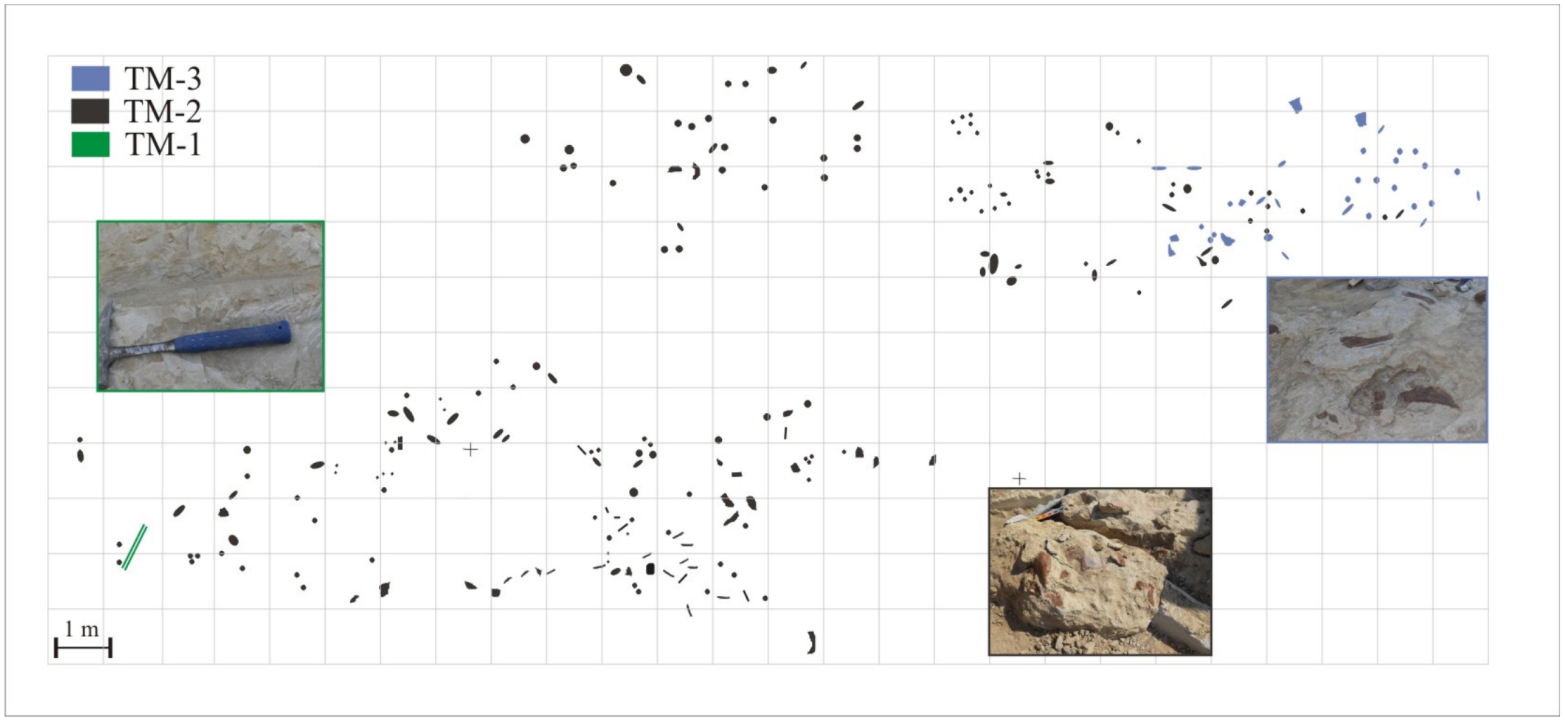
Map of the fossil-bearing site at Velaux-La Bastide Neuve. Two zones were explored, during the two field campaigns (2009 and 2012) on a total surface of about 140 m^2^. The three taphonomic modes (TM) are represented by three colors—TM1 in green, TM2 in black, and TM3 in red—, which represent the three successive deposits.

The position of each bone collected during the 2009 and 2012 field seasons was mapped (Fig 3). Data were also collected as to the nature, dimensions and orientations in the case of elongate bones. The fossil-bearing area covers a surface of about 140 m^2^. Features of the bones (weathering stage, fractures) were studied in laboratory (University of Poitiers and IRSNB), in order to document the taphonomy of the locality. Stereonet free program was used for drawing bidirectional rose diagrams and the nonparametric Rayleigh’s test was performed for testing uniformity of the data.

## Results

### Magnetostratigraphy

**Fig 4:**
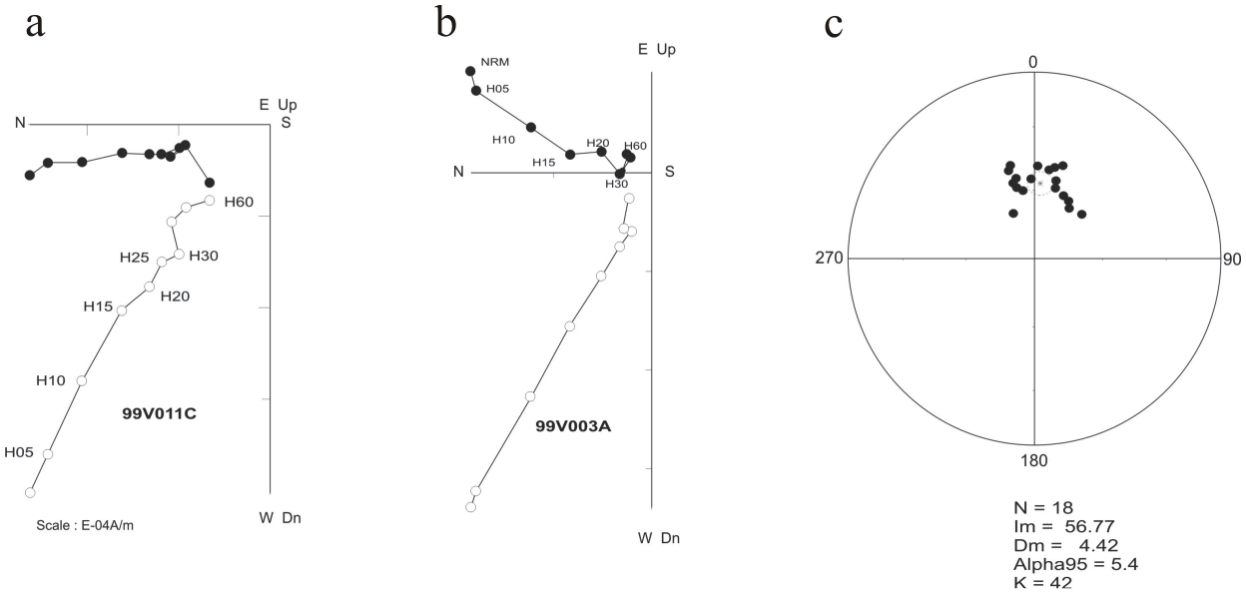
Zijderveld diagrams. (a) and (b) representative Zijderveld diagrams of the samples (c) equal-area stereographic projection of characteristic direction.

Concerning the magnetostratigraphy, only one paleomagnetic component could be recognized in the studied samples (Fig 4a and 4b). The greater part of remanent magnetization was removed at 60 mT, which may indicate that magnetite is the main carrier. The average directions were determined after tilt correction (Fig 4c): the mean direction of the characteristic components is (I = 56.7, D = 4.42, a = 5.4, kappa = 42; n = 18). The mean direction is different from the present day magnetic field at the sampling site suggesting a primary remanence of magnetization. The rocks exhibit a single normal polarity. According to the biochronological data, the correlation with the Geomagnetic Polarity Time Scale (GPTS) could be done with the normal chron of the chron C32 (C32n.1n, C32n.2n or C32r.1n). Accordingly, an age of between 70.9 and 73.3 Ma, or an adjusted age of between 71.6 and 74.0 Ma [12], can be assigned to the Velaux-La Bastide Neuve section.

### Section, Sequences, and Facies

The studied section has a thickness of 16.3 m from the basal level located below the lowermost bone-bed to the top of the upper limestone bed. The section can be divided into nine depositional sequences (Fig 2). The lower contact of each sequence is erosional and each sequence fines upward. Four sequences are characterized by the same facies succession: sandstones at the base followed by calcareous siltstones. Only the uppermost sequence contains limestones, indicating a different sub-environment.

Oxidized glauconite grains were found in sandstones from sequence 3 (samples VBN-18 and VBN-20A) and were dated using K-Ar analysis. Results provide ages of 122.2 ± 3.2 Ma and 123.6 ± 3.4 Ma (Table 1), corresponding to the early Aptian stage [12], [13].

**Table 1:** Radiometric results on glauconite grains belonging to two sandstone samples.

Sample	K (%)	^40^Ar rad (nl/g)	^40^Ar air (%)	Age (Ma)
VBN-18	1.68	8.073	37.5	122.2 ± 3.2
VBN-20A	1.69	8.265	48.3	123.6 ± 3.4

**Fig 5:**
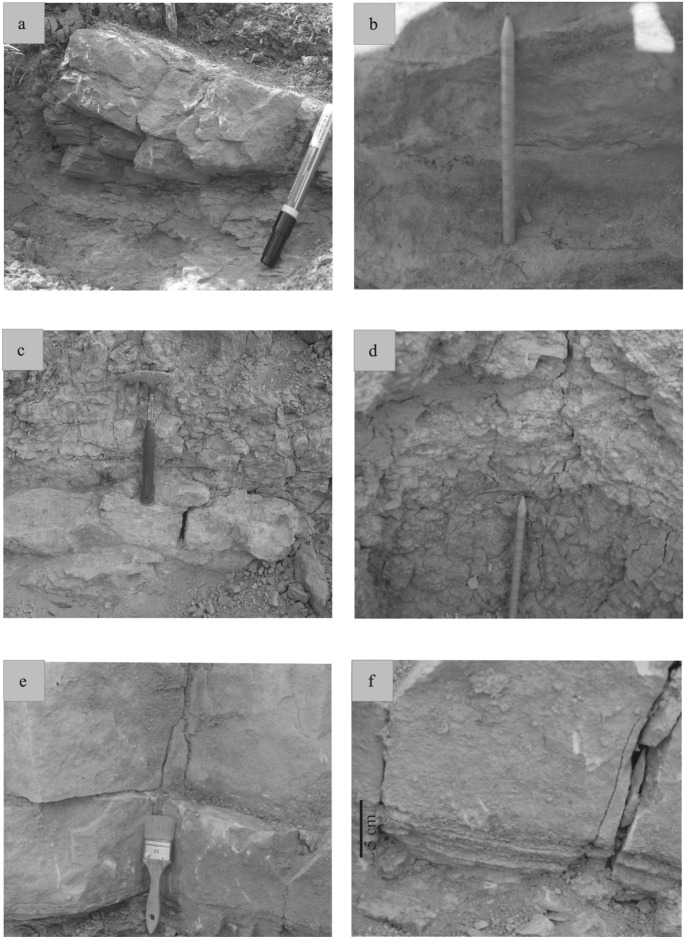
Lithofacies. (a) small-scale cross-laminated sandstones (seq. 1) (b) beige fine-grained sandstones showing no sedimentary features (seq. 3) (c) variegated siltstones overlying grey sandstones (seq. 6) (d) blue-grey siltstones overlaying variegated siltstones (seq. 7) (e) both first beds from the massive limestone of the sequence 8 (f) thin muddy layers below the lower limestone bed, and the undulated limestone layer at the base of the lower bed (seq. 8).

**Fig 6:**
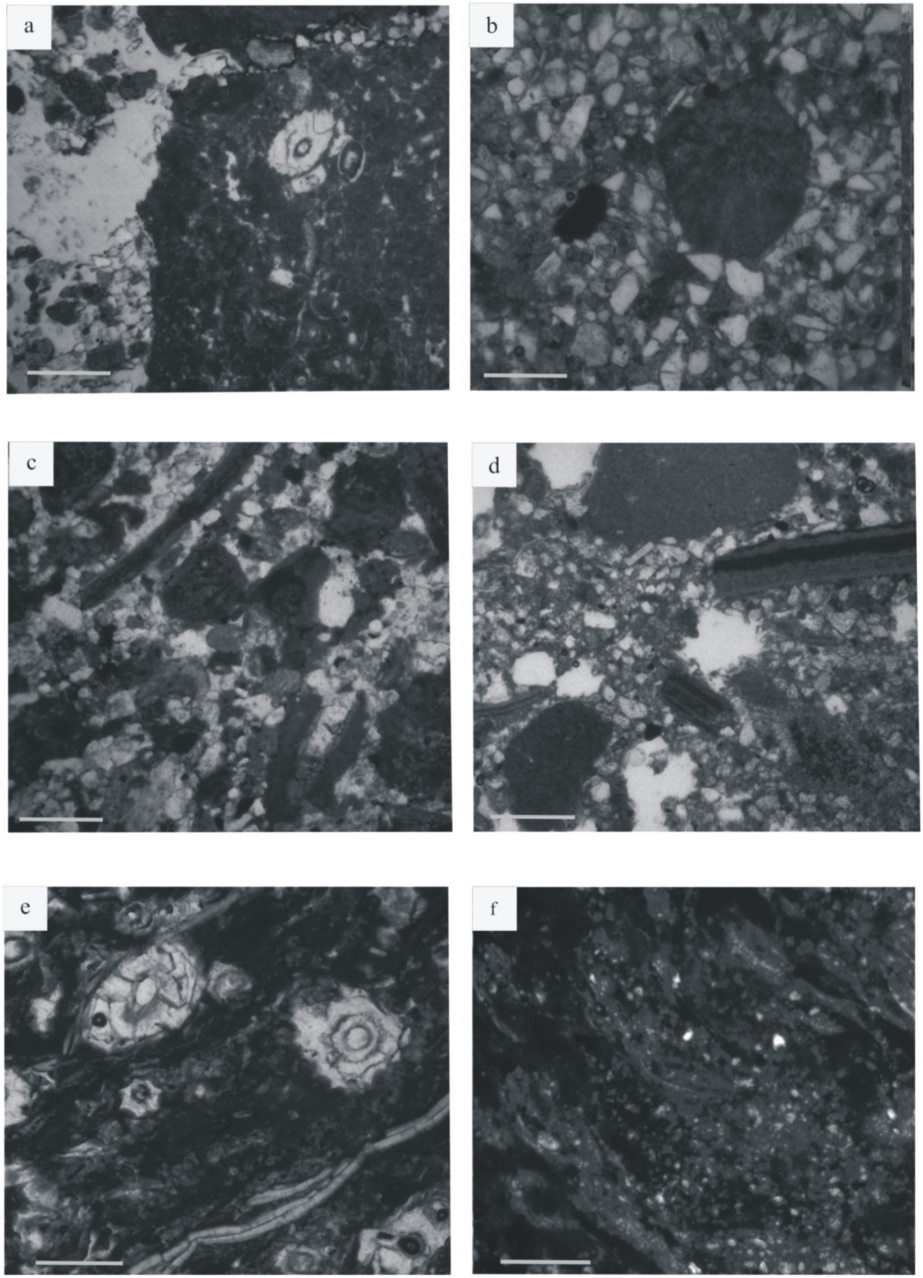
Microfacies. (a) MF1 –sandy conglomerate including mollusk valves and micritic grains (b) MF1 –carbonated intraclast including *Microcodium* (c) MF2—fine-grained sandstone with algal ball and mollusk shells (d) MF3—coarse-grained siltstone including micritic grains and mollusk shells (e) MF4—micritic limestone including *Microcodium* and shells (f) MF5—fine-grained siltstone coated with iron oxides. Scale bars = 500 μm.

**Fig 7:**
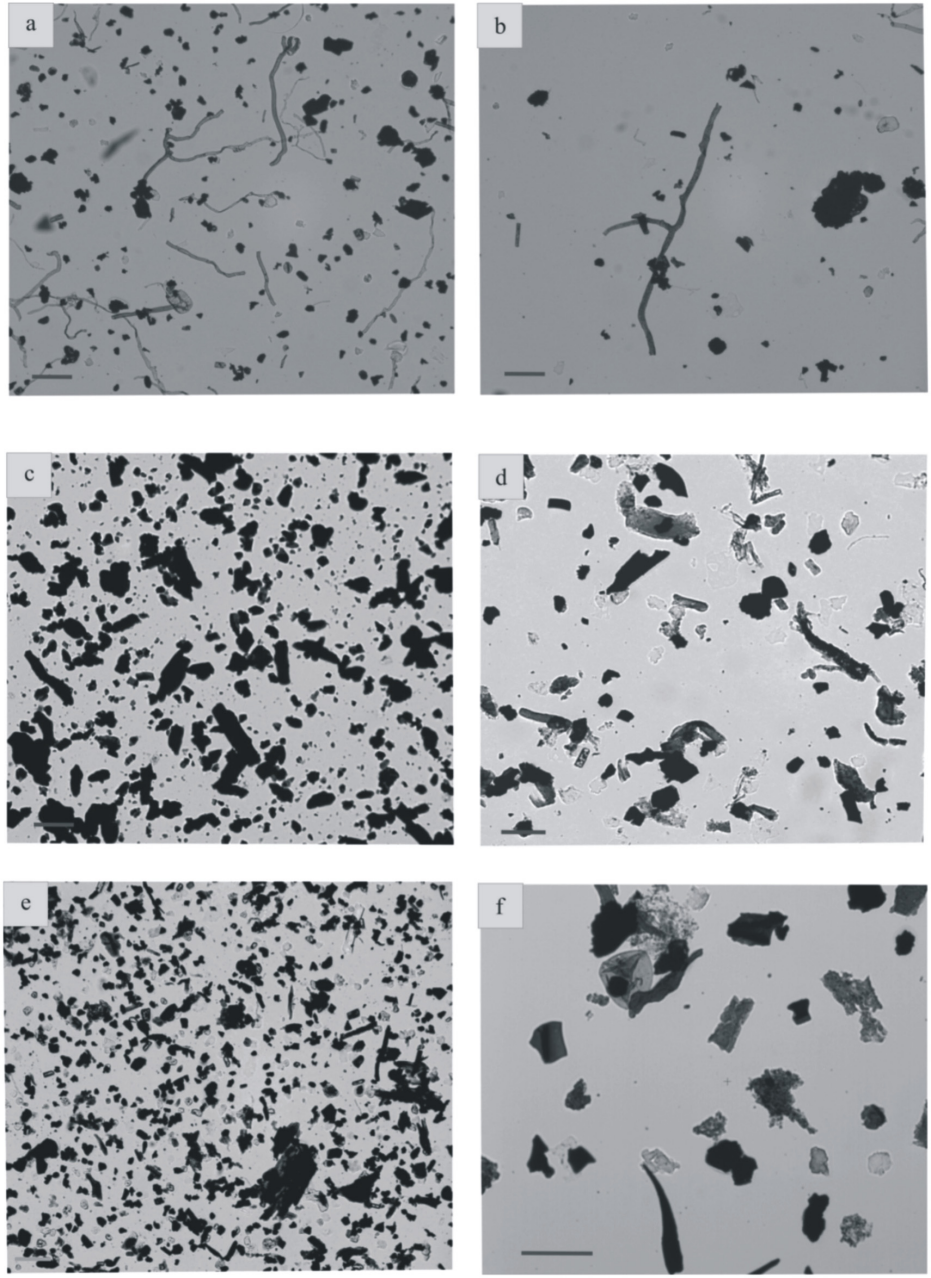
Palynofacies preparations from the Velaux-La Bastide Neuve sedimentological succession. (a) PF1- high diversity section with lignitic debris, charcoal and abundant fungal hyphae (scale bar = 100 μm) (b) PF1- fungal hyphae showing septae, lignitic debris, charcoal and amorphous organic matter (scale bar = 50 μm) (c) PF2- numerous rectangular and rounded charcoal grains, and lignitic debris (scale bar = 50 μm) (d) PF2- A.O.M., charcoal and pollen grain (scale bar = 50 μm) (e) PF3- charcoal and tracheid fragments (f) PF3- fungal spore, diverse plant debris (e.g. tracheid fragments), charcoal and A.O.M. (scale bar = 50 μm).

The entire succession comprises five dominant lithofacies: sandstones (Fig 5a and 5b), siltstones (Fig 5c and 5d), limestones (Fig 5e and 5f), mudstones (Fig 5f) and lignite layers. The sedimentological section is dominated by variegated siltstones (52% of the section thickness) and sandstones (34%; Fig 2). There are five microfacies: bioclastic micro-conglomerate (Fig 6a), bioclastic sandstone (Fig 6b), bioclastic siltstone (Fig 6c and 6d), micritic wackestone (Fig 6e) and azoic oxidized siltstone (Fig 6f). Palynological analysis allow discrimination of three palynofacies: a high diversity palynofacies (Fig 7a and 7b), a charcoal-rich palynofacies (Fig 7c and 7d) and a phytoclast-rich palynofacies (Fig 7e and 7f). Lithofacies are associated with microfacies and palynofacies to specify the nature of sub-environments. Nonetheless, some of the same microfacies and palynofacies are associated with different, but related, sub-environments.

#### Facies 1

**Fig 8:**
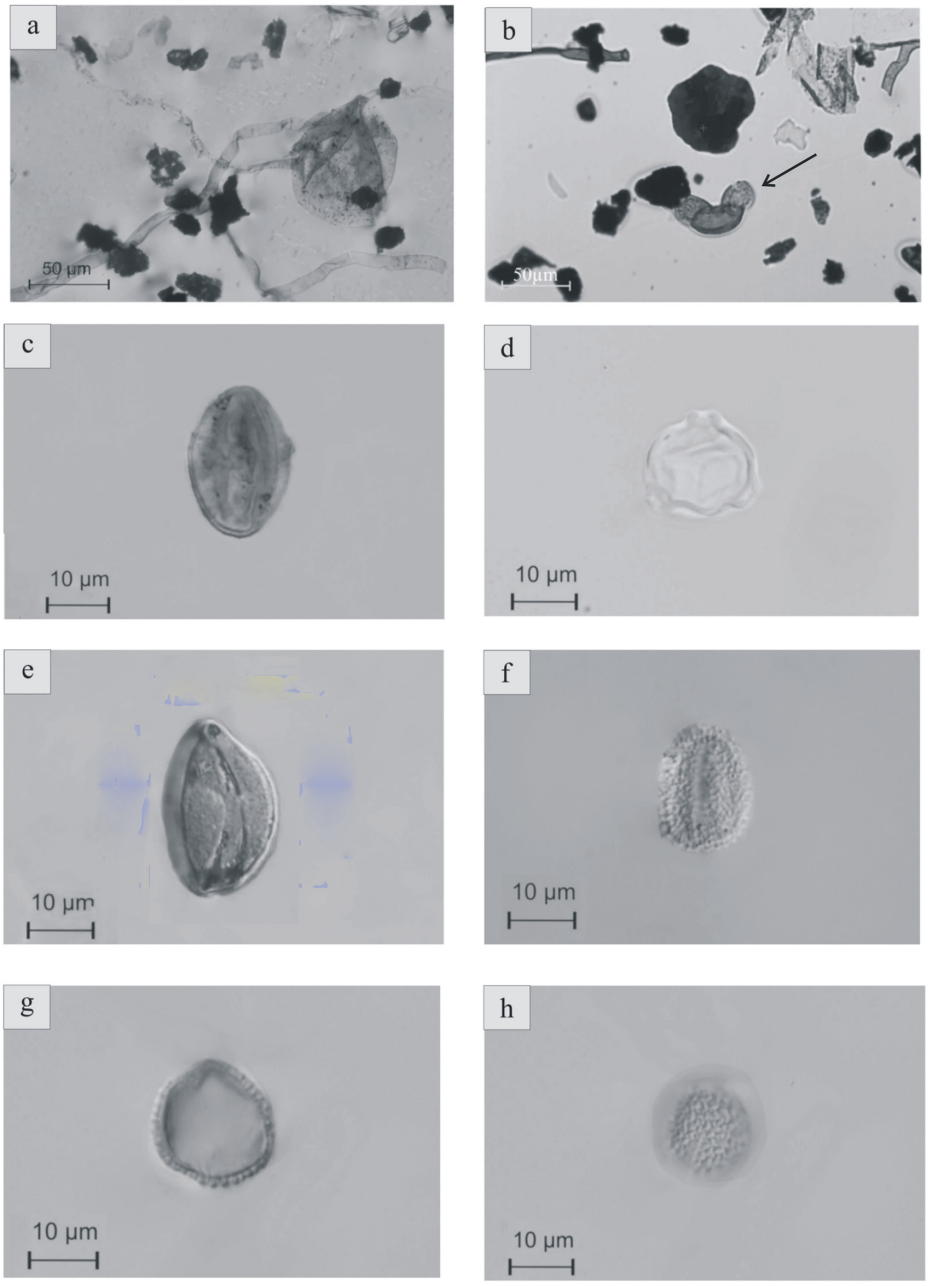
Fungal hyphae, spores and palynomorphs. (a) fungal spore with thin wall and folds and connected to an hypha (*Paleomycites*?) [12-VBN-05; sequence 1] (b) bisaccate pollen grain among coalified plant debris and hyphae (arrow) [12-VBN-05; sequence 1] (c) prolate tricolporate pollen grain in meridian view (d) pollen grain probably belonging to the Normapolles complex (polar view) (e) monoaperturate pollen grain, probably monosulcate pollen grain (f) semitectate, reticulate, probably monosulcate pollen grain (g) and (h) monosulcate pollen grain; same taxon in median (g) and high (h) focus.

Facies 1 corresponds to predominantly sandstone deposits that in all cases overlie an erosional base (Fig 5a). The facies is composed of yellow to grey sandstones and displays various grain sizes, from conglomeratic to coarse-grained sandstones. Bed thickness varies from 5 cm to 80 cm. Sandstones exhibit small-scale planar cross-stratification and horizontal lamination. At the base of sequence 7 (Fig 2), a thin clast-supported intraformational conglomerate is present. Sandstones are in some cases interstratified with thin layers of lignite. Sandstone abundance decreases from the base of the section to the top. Thin black lignite laminae are interstratified within sandy beds, especially at the top of sequences 2 and 3 (Fig 2). Fossil trunk wood traces are also found in these layers. Facies 1 comprises two distinct microfacies. The first one is the coarsest, represented by a micro-conglomerate composed of subangular to subrounded quartz and feldspar grains. It contains micritic grains as algal balls, glaebules, peloids, calcareous intraclasts (Fig 6a), fragments of mollusk shells (Fig 6b), “algae” and foraminifera. The second microfacies is a bioclastic sandstone that contains the same skeletal elements but is matrix-supported. The palynological assemblage comprises abundant phytoclasts (rounded charcoal grains and various lignitic debris), bisaccate pollen grains, fungal spores (Fig 7a) and fungal hyphae (Fig 8a).

#### Facies 2

The deposits of facies 2 are characterized by medium to coarse-grained sandstones (Fig 5b). The sandstones are in some cases variegated, contain mollusk shells, calcareous nodules and, locally, orange mud clasts. Sediments are mixed with reworked debris, including marine invertebrate fossils (foraminifera and “red algae”). Feldspar grains, foraminifera, *Microcodium* (calcite prisms induced by biogenic processes [14], [15]), freshwater fossils as Characeae gyrogonites, algal balls, mud coated grains and stromatolites are also present in these deposits. Skeletal grains are in some cases highly micritized and not easily identifiable (Fig 6c). The palynofacies contains the same palynomorphs as facies 1; including phytoclasts, abundant charcoal and bisaccate pollen grains (Fig 8b), fungal hyphae and fungal spores.

#### Facies 3

The third facies consists of variegated and fine-grained siltstones. The siltstones are bioclastic—and contain mollusk shells, foraminifera, *Microcodium*, algal balls, mud-coated grains and stromatolites (Fig 6d). Skeletal grains are highly micritized. The palynological material constitutes mostly phytoclasts, mainly with charcoal and lignitic debris (Fig 7e); but fungal hyphae are also present. Pollen grains (Fig 7f) are rare. Charcoal grains are less abundant than in the facies 1. Tracheid fragments are bigger and better preserved than charcoal.

#### Facies 4

Sediments of facies 4 are variegated, but mostly reddish and fine-grained siltstones. No sedimentary structures are present in these siltstones, but pedogenic features, such as carbonate nodules are well developed. Skeletal elements are also lacking (Fig 6f). There are only rare reworked glauconite grains, and mineral grains are coated by iron oxides. The corresponding palynofacies contains nearly the same organic particles as facies 1 and 2, although fungal hyphae (Fig 7c) are absent. Charcoal grains are very abundant and lignitic debris is less common although less degraded than in facies 3. A few bisaccate (Fig 8b), tricolporate (Fig 8c) and monosulcate (Fig 8e) pollen grains, and also some Normapolles (Fig 8d) occur.

#### Facies 5

The fifth facies is only present in sequence 8 (Fig 2) and consists of resistant limestone beds interstratified with thin greyish mudstones. The limestones reach 1.2 m in thickness, and include very fine-grained beds containing mollusk shells and ostracod valves. Undulatory laminae are present at the base of the lowermost bed (Fig 5f). Upper beds are massive without any sedimentary structure. Very few mudstone layers occur in this facies; where present, they are horizontally laminated. Limestones are overlain by reddish siltstones presenting color mottling. The microfacies is a fine micritic limestone, containing abundant *Microcodium* (Fig 6e) but also charophyte gyrogonites and some ostracods. *Microcodium* are in some cases wrapped in thin mud layers. No detrital grains are present in this facies, but only a fine micritic mud with a wackestone texture including *Microcodium* features and shells dispersed in a micritic matrix.

### Taphonomy

#### Taxonomic and skeletal composition

**Fig 9:**
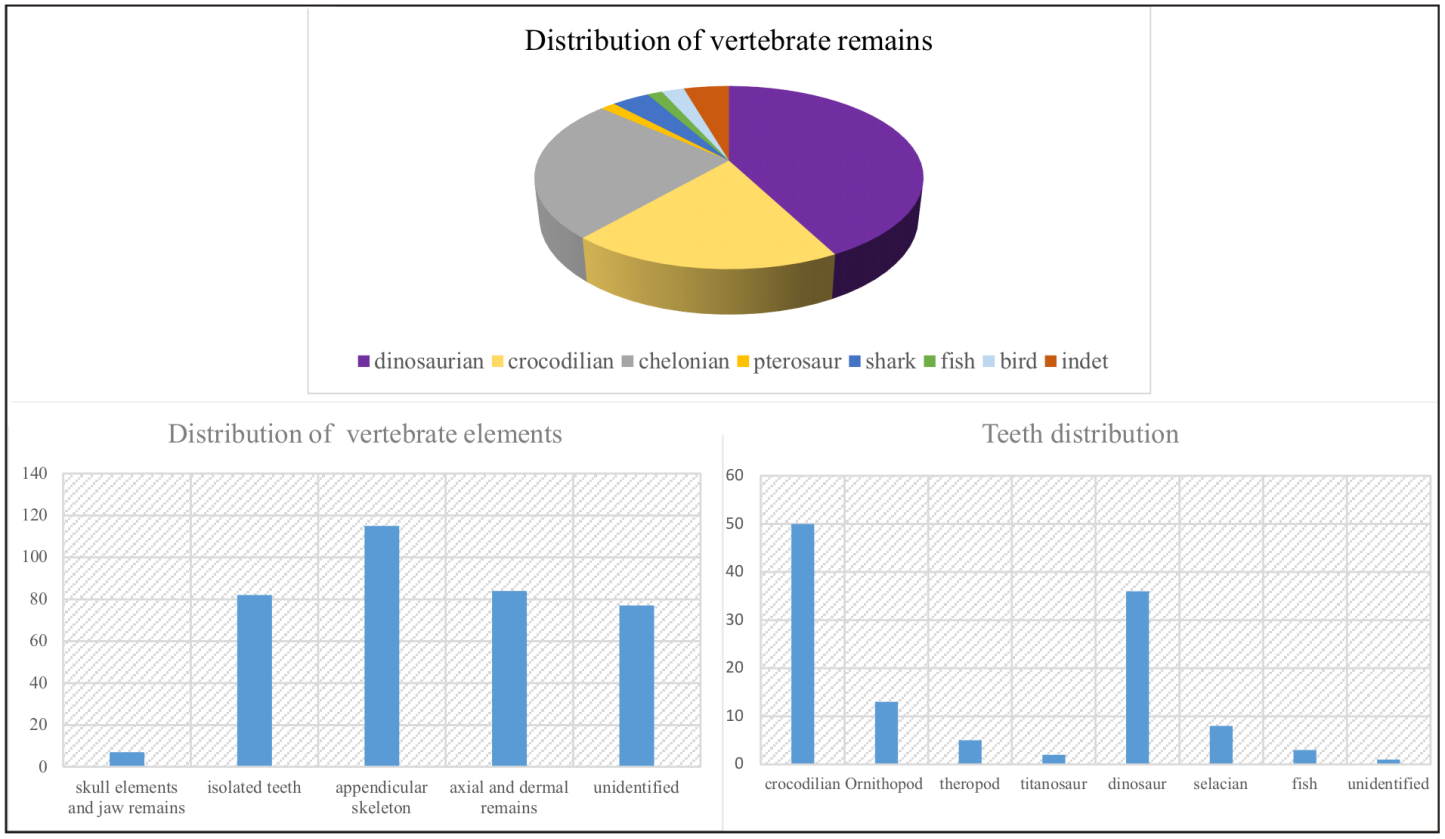
Vertebrate remains distribution and bone nature. Dinosaurs and chelonians dominate the fossil assemblage. Elements as teeth or plates are particularly abundant. Data were taken during two field campaigns, in 2009 and 2012.

Three hundred and eight vertebrate fossils—including bones, plates, osteoderms, and teeth—belonging to several taxa were inventoried at Velaux during the two field-campaigns. Dinosaur remains dominate the vertebrate fossils (Fig 9—above), representing over 38% of the total assemblage collected during the 2009 and 2012 campaigns. Crocodilian elements are particularly abundant (16%), and mainly represented by shed teeth (Fig 9—below right). Numerous crocodilian (non-shed) teeth occur together with incomplete juvenile skulls and one complete adult skull (MMS/VBN-12-10A). Chelonian carapace and plastron parts are abundant (64 specimens collected so far), representing 22% of the assemblage. Pterosaur, hybodont shark and fish elements are also represented in the assemblage although they are rather rare (17, 10 and 4 elements, respectively). Among the identifiable dinosaur remains, ornithopod (39%) elements are the most abundant. Various ankylosaur (24.5%) elements were found including osteoderms, long bones and one scapula. Titanosaur elements (22.4%) comprise vertebrae and long bones. Theropods (14%) are mainly known from isolated teeth, with few long bones and vertebrae. Small fossils are numerous, dominated by teeth and chelonian shell parts. Fragile bones such as ribs, chevrons or cranial elements are under-represented (Fig 9—below left).

#### Accumulation modes

The vertebrate fossils of the Velaux-La Bastide Neuve site occur in three thin layers with different accumulation modes reflecting different depositional histories. Investigations were concentrated on the three vertebrate-bearing layers (Fig 3), located in the lower part of the sedimentological section in sequences 1, 2 and 3 (Fig 2). Bones are particularly abundant in the second fossiliferous layer, which consists of fine to coarse-grained sandstone.

Isolated and disarticulated elements belonging to a single taxon were found in variegated coarse-grained siltstones from sequence 1, and were interpreted as an overbank facies. Two long (>2m) fragile but very well preserved parallel bones were found in 2009 in these variegated siltstones, in association with skin impressions. These elements can be identified as sauropod cervical ribs (Koen Stein, pers. comm.). The lowermost fossiliferous layer cannot be regarded as a bone bed, in the current state of excavation, as all the elements possibly belong to a single individual [16].

**Fig 10:**
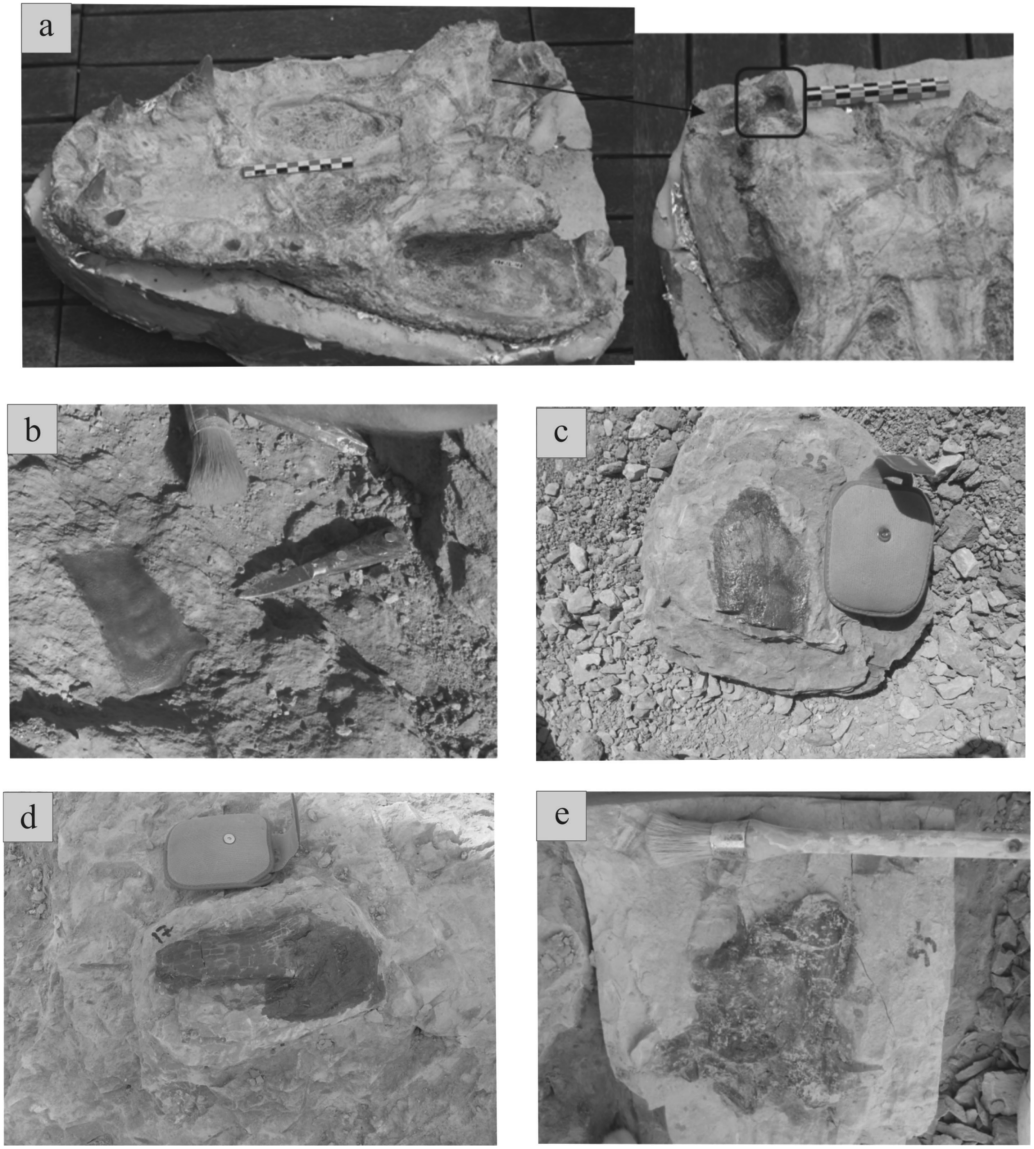
Plate illustrating the diversity of vertebrate remains collected from the two bone beds of Velaux-La Bastide Neuve. (a) complete crocodilian skull (MMS/VBN-12-10A) from an adult specimen that shows a deep tooth pit near the ocular fenestra (square) (b) ornamented chelonian plate attributed to the genus *Solemys* (c) non-ornamented chelonian plate attributed to the genus *Polysternon* (d) indetermined dinosaur phalanx (e) dinosaur vertebra.

**Fig 11:**
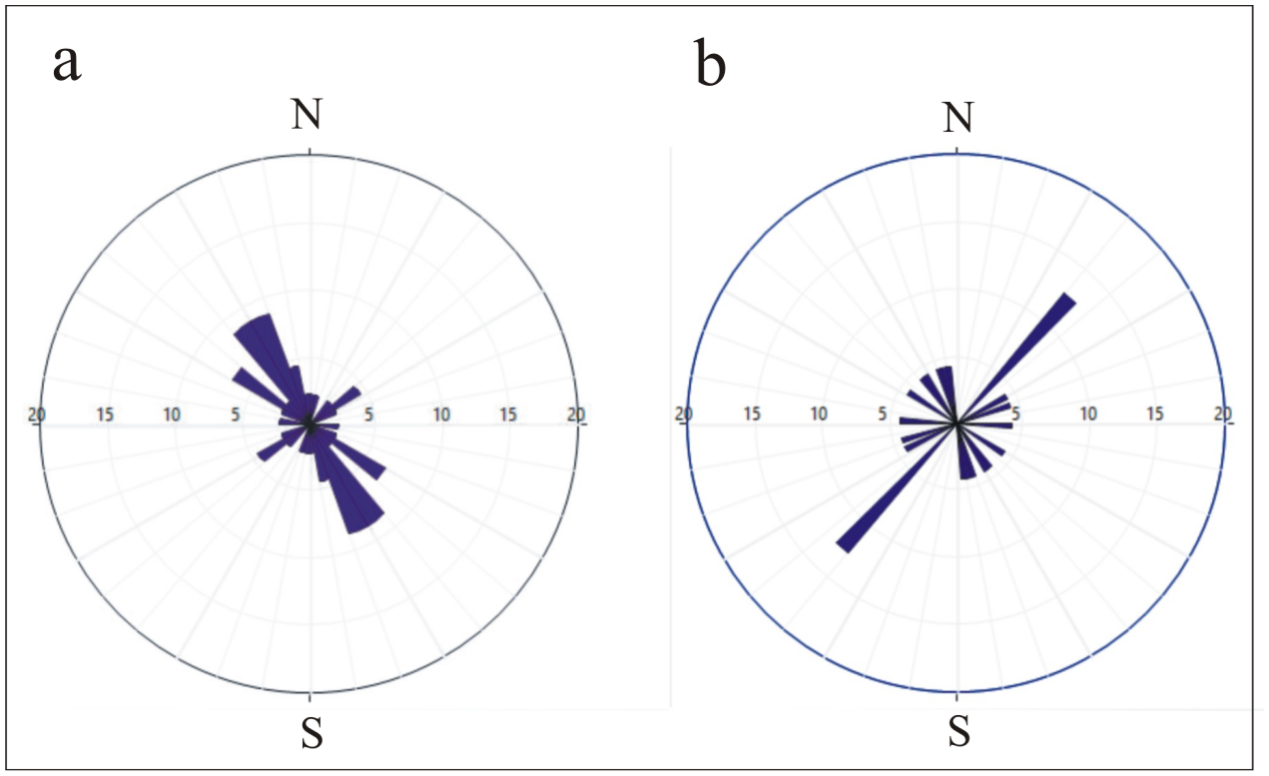
Rose diagrams showing the long-axis orientation for bones collected at Velaux-La Bastide Neuve. (a) represents the south-east/north-west trend for long bones from the second taphonomic layer (BB1; n = 23); and (b) shows the orientation pattern for long bones of the third taphonomic layer (BB2; n = 12). Statistical analyses show that the null hypothesis of uniformly distributed data can be rejected for (a) but cannot be rejected for (b); Rayleigh’s test, (b) z = 2.969, p < 0.05; (b) z = 0.661, p > 0.05.

The second fossiliferous layer is represented by a multitaxic accumulation of elements included in coarse-grained conglomeratic sandstones from sequence 2. The following elements are represented in this multitaxic bone bed; ornithopod bones and teeth, bones and teeth from the titanosaur *Atsinganosaurus velauciensis* [6], theropod teeth, crocodilian teeth, isolated bones, skulls (Fig 10a) and one osteoderm, chelonians carapace and plastron elements and long bones belonging to two different taxa—*Solemys* (Fig 10b) and *Polysternon*-, hybodont shark teeth, fish teeth and pterosaur bones. Most of bones are disarticulated, except partially articulated titanosaur skeletons [6]. Elements, presenting various sizes and shapes, are mixed together and are often entangled in the sandy matrix. Long bones in this layer tend to be aligned along a NW-SE axis (Fig 11). Predation marks are extremely rare, they are even absent on titanosaur bones, although crocodilian shed teeth are rather abundant. A single deep tooth mark is present near the ocular fenestrae of a complete adult crocodilian skull discovered in 2012 in this bone bed (Fig 10a). Trample marks have not been identified in this material; these are characterized by shallow, sub-parallel scratch striae on the bone surfaces [17], [18].

A third taphonomic mode consists of disarticulated bones (Fig 10d and 10e), plates, and abundant teeth found in sequence 3. The bones are more dispersed than in the other modes and belong to several taxa. Ankylosaur long bones and osteoderms are only present in this silty sandstone layer. Theropod teeth, ornithopod teeth, crocodilian osteoderms and teeth, and chelonian plates belonging to both *Solemys* and *Polysternon* (Fig 10c) occur. Neither predation marks nor tramples marks are present. The chelonian remains are very well preserved and usually less degraded than dinosaur bones. Long bones (mostly belonging to the ankylosaur) are mostly oriented NE-SW but other elements present orientations that are quite varied (Fig 11).

## Discussion

### Paleoenvironment: integrated litho-, micro-, and palynofacies

The observed lithologies, microfacies and palynomorphs documented in the nine depositional sequences exposed at Velaux-La Bastide Neuve indicate several alluvial sub-environments, ranging from channel lag to floodplain.

#### Facies 1

Conglomeratic beds (e.g. sequence 2) correspond to the basal deposits in a stream channel, whereas all finer sandstones are interpreted as channel levee deposits or crevasse splay deposits. The presence of small-scale planar cross-stratification indicates low-energy sedimentation [19], [20], whereas horizontal bedding corresponds to upper flow regime conditions [19], [21]. Mud clasts could indicate reworking of channel banks within the river [22] or basal scour during channel incision. Lignite layers are interpreted as organic matter accumulations introduced by bank erosion into the river channel, collected by the river stream and then deposited along with bedload sand during lower flow regime conditions [22]. Glauconite grains dated from 122.2 ± 3.2 Ma and 123.6 ± 3.4 Ma are interpreted as reworked grains probably originating from Aptian marine limestones, widespread in this area (e.g. [23]). Foraminifera observed in thin sections are also probably reworked from older carbonate rocks. *Microcodium* [15] may also be reworked. Broken and rounded grains occur in the upper flow regime sediments, resulting from deposition in the channel or in a crevasse play. Phytoclasts and in particular lignitic debris reflect the presence of vascular plants in the environment, either from riparian forests (if found in channel deposits) or other remote forests. The roundness of most charcoal indicates a prolonged transportation of these particles in the river channel. Fungal hyphae indicate recycling of wood debris from surrounding soils [24].

#### Facies 2

Facies 2 is interpreted as levee deposits. Because feldspar crystals are unstable and quickly weathered, their presence in the sandstones reflects rapid transport and deposition from the sediment source (e.g. [25]). The micritization of many skeletal elements reveals shallow water conditions [26], [27]. The rounded shape of charcoal still indicates a prolonged transportation from the plant source. These charcoal grains potentially reflect different stages of organic matter maturity. Indeed, they are common features in palynological assemblages, corresponding to charring states of diverse organic debris; they may be a result of wild fires. Vegetation in levee facies was likely abundant, suggesting rare inundation episodes [28].

#### Facies 3

The presence of color mottling, in addition to the absence of stratification, in the fine-grained siltstones of facies 3 suggests soil formation, resulting from periods of subaerial exposure (e.g. [29], [30]). The paleosol profiles are particularly rich in carbonates (Table 2). Broken mollusk shells and other skeletal grains indicate that they were transported by relatively high currents before deposition within fine sediments. Moreover, mud coated grains reflect rather shallow water conditions [26], [27]. Charcoal grains are less abundant than in the previous facies. The large size of tracheid fragments suggests better preservational conditions, and/or the proximity of source plants to the depositional site, and/or low hydrodynamic conditions. The presence of both broken skeletal elements and well preserved phytoclasts in fine-grained sediments indicate an intermediate sub-environment, intermediate between the active channel and surrounding floodplain. Therefore, this facies is tentatively interpreted as a proximal floodplain environment.

**Table 2:** Carbonate concentration of paleosol samples. The table displays the CaCO_3_ content of calcareous siltstones. All samples are particularly rich in carbonates. See Fig 2 for correlation with these samples.

Sample	VBN-01	VBN-02	VBN-22	VBN-23	VBN-25	VBN-27	VBN-28	VBN-31	VBN-32	VBN-33	VBN-41
CaCO_3_ (%)	**23.8**	**24.3**	**48.5**	**93.4**	**38**	**30.9**	**34.1**	**50**	**32.7**	**29.7**	**38.1**

#### Facies 4

Facies 4 is the finest grained, consisting predominantly of siltstones and mudstones. It is interpreted as a low-energy alluvial plain facies rather far from the active channel, according to the size of the grains, the oxide-coated grains, the quantity of mud and the lack of bioclasts. Deposits are variegated, probably indicating local changes from oxidizing to reducing conditions [29]. The silty sediments were exposed subaerially and subject to soil-forming processes leading to paleosol formation in a well-drained floodplain as it is shown by mottled sediments, and calcareous nodules (e.g. [31]). The mudstones are horizontally laminated, and are interpreted as a result of settling of suspended sediment in overbank areas during flood events [32]. The low amount of lignitic particles in palynological assemblages, although they are well preserved, suggests long-range transportation from the source.

#### Facies 5

Undulatory laminae at the base of the lowermost limestone bed (Fig 5f) suggest deposition in a shallow lake with bottom currents [19]. Upper beds are massive without sedimentological structure. Because of its very fine texture and the presence of ostracods, this calcareous facies may be interpreted as freshwater limestone deposits within an alluvial setting [33]. Lake formation could have resulted from avulsion when channels were abandoned [18]. During periods of high discharge, flows can spread out onto the floodplain and then fill abandoned channel to form a lake [34]. On the other hand, such a lake could have been formed independently from the fluvial system. Absence of any form of sedimentary structure in the upper carbonate beds probably represents low-energy lake sedimentation with high biogenic productivity [19], [31]. These micritic limestones correspond to autochthonous deposition of carbonate mud. The microfacies reveal very fine matrix and the presence of freshwater “algae”, mollusk shells and ostracod valves [35]. The fine-grained sediments reflect a very calm depositional environment without channel sediment contribution. Moreover, the presence of fossils and the homogeneous texture of the lacustrine wackestone indicate a shallow low-energy depositional context [36], [37], as well as the decrease in the detrital input.

### Taphonomy

The vertebrate assemblage collected in 2009 and 2012 includes two chelonians, *Solemys* and *Polysternon* [38], which are freshwater taxa that lived in fluvial environments [39], [40]. Chelonian specimens are abundant within the different taphonomic modes and are usually well preserved (Fig 9), suggesting a local source for the turtle carcasses. The crocodilian skull and a mandible probably belonging to a basal Alligatoroïdae are similar to a specimen from this crocodilian family [41] previously found in Romanian pond deposits, suggesting that they lived in lacustrine habitats [40]. The excellent preservation of several crocodilian specimens in fluvial deposits from the Velaux-La Bastide Neuve locality also indicates short transport followed by a rapid burial at the local site and suggests that this crocodilian could also live in fluvial environments. The presence of teeth of the hybodont shark *Meristonoides* (Gilles Cuny, pers. com.) is rather unusual. However, hybodont sharks are abundant in freshwater environments during the Cretaceous, although it is difficult to determine precisely if they lived only or occasionally in freshwater environments [42].

Predation marks are scarce: only one supposed predation mark is observed among the total assemblage. Tooth marks on dinosaur bones are usually attributed to scavenging and prey carcass utilization [43], [16], and [21]. If so the scarcity of such marks on the bones of Velaux-La Bastide Neuve assemblage might reflect a low incidence of scavenging at the locality. Alternatively it may also be hypothesized that prey were particularly numerous and that scavengers only ate the fleshy part of carcasses [44] or preferred some portions of the skeletal remains [45]. Tooth-marked bones are never frequent in dinosaur localities and that theropod dinosaurs did not routinely bite bones during prey carcass utilization [21].

*Post-mortem* fractures are frequent in the fossil assemblage, but bone edges usually remain sharp, indicating that the transport that affected the dead carcasses was rather short [43]. However, numerous parallel transverse fractures (perpendicular to bone fibers) represent *post-mortem* fracturing that affects bones during burial compaction [46], while longitudinal breakages represent weathering, a pre-diagenetic process [16]. Oblique and in rungs breakages are also common and consist of pre-fossilization fracturing, revealing fresh bone damage [44].

Perthotaxic features (bone modification processes active on the land surface [47]) are not found on the bones inspected here. Weathering of bone surfaces is weak in the assemblage. This indicates that the bones were not exposed subaerially for any significant time before or after reworking and that they were quickly buried. The chelonian remains are very well preserved and usually less degraded than dinosaur bones, suggesting that their transport was of shorter duration and that they lived directly in (or very close to) the area.

Most of vertebrate remains are very well preserved in the three fossil-bearing layers. The exceptional preservation of two parallel sauropod cervical ribs, associated with skin impressions in the lowermost layer, reflect rapid burial [48] in fine-grained sediments after short transport in the alluvial plain. Associated elements (partially articulated titanosaur skeletons) from the lowermost bone bed are not complete suggesting that the rest of the skeleton was dissociated earlier.

The taxonomic diversity of the two bone beds suggests distinct accumulations of vertebrate remains belonging to individuals that died in different places and/or at different times. Their remains were then transported by river streams from their different habitats, deposited and finally gathered in the same area in a channel.

It appears that the lowermost bone bed presents a non-random orientation pattern with a major south-east trend for the elongate elements (Fig 11). In this sandy layer elements are heterogeneous with various sizes and shape ranges. This orientation pattern with heterogeneous elements results probably from larger elements acting as obstacles for the other, smaller, elements [49]. No clear preferential orientation in the uppermost layer probably results from low-energy depositional conditions. In this case, currents were probably too weak to orient all the elements in the same direction. The apparently random orientation in this layer could also originated in the abundance of elements or by their sudden deposition. It has been demonstrated that bones transported in shallow water adopt a parallel orientation to the flow, suggesting a NE-SW direction for the currents that transported these bones [49].

### Sedimentology and taphonomy

Sedimentology and taphonomy, together, permit a reconstruction of the paleoenvironments in Velaux-La Bastide Neuve locality. Interpretations resulting from both sedimentological and taphonomic studies, indicate that the bones were deposited in a fluvial environment. The size of the grains, related to downstream decrease in discharge, is a good indicator for assessing the distance between the depositional environments from the main stream channel. Coarser sediments (conglomeratic and sandy facies) deposited close to the channel, were accompanied by heavier vertebrate elements. Finer sediments were deposited further in the alluvial plain, with lighter elements. Sandy fossil-bearing deposits are interpreted as either channel or crevasse splay facies, while silty deposits are interpreted as overbank facies.

Organic particles are typically found in the finer sediments because of their low densities. Thus, organic matter reflects a low energy depositional environment far from the river stream.

Numerous charcoal fragments might indicate the presence of wildfires in the paleoenvironment. Moreover wildfires might intensify landslides and thus influence sedimentation [50]. On the other hand, charcoal might also be the result of plant debris maturation. Charcoal is common in palynological assemblages. Palynofacies therefore confirm the results of the sedimentological studies. Preservation of charcoal, lignitic debris and other organic particles probably reflect flooding events that concentrated phytoclasts. The scarcity of pollen grains in the sediment is more likely the result of the distance from the vegetation sources than their non-preservation.

The vertebrate assemblage discovered at Velaux-La Bastide Neuve clearly represents a mixture of aquatic and terrestrial animals that lived in different habitats. Transported vertebrate carcasses appear to be well preserved both in channel-fills and overbank deposits, due to rather short transport in a low-energy fluvial system. Chelonians and crocodilians were potentially (para) “autochthonous”—their carcasses experienced very little transport inside their life environment-, whereas dinosaurs and sharks were “allochthonous”: the latter did not live directly inside the fluvial realm, but in its vicinity and their remains were transported.

During flood events, the river water could overflow channel banks and spread out over the alluvial plain. The finest deposits result from settling of material further from the active channel and accumulated in overbank areas. The river system is interpreted to have had a broad floodplain subjects to periods of soil development. Major vertebrate taxa (dinosaurs, turtles, pterosaurs and crocodiles) lived in this fluvial environment. Palynological analysis confirm that this environment was surrounded by flowering plants and conifers. Repeatedly rainfall could induce flooding events, which resulted in transport of vertebrate remains and organic matter within the system. Different sub-environments then succeeded in the same area and the sedimentological facies in Velaux record these fluvial deposit successions.

## Conclusion

The sedimentological record observed in Velaux-La Bastide Neuve site represents a vertical succession of five facies that are interpreted as channel and floodplain deposits in the medial part of a fluvial depositional system; moreover, the section shows evidence for both river and lacustrine environments. There are nine sedimentological sequences dominated by overbank deposits representing successive flooding episodes. Each sequence is characterized by erosive and depositional episodes. Relatively fine-grained sediments and amalgamation of channel-fills suggest a river system surrounded by a floodplain. Lack of floodplain deposits overlying the sandstones in sequences 2 and 3 suggest several episodes in the river channel down cutting within this zone.

Palynological study corroborates the environmental model proposed, confirming relatively low hydrodynamic depositional conditions and dry climate alternating with wet periods. Vascular plants were present in the paleoenvironment. Palynological assemblages show that angiosperms were also represented in the environment. A few triaperturate pollen grains possibly belonging to Normapolles complex indicate that the vegetation from the Velaux region was probably composed of both gymnosperms (Coniferales) and angiosperms. Transported vertebrate carcasses appear to be well preserved both in channel-fills and overbank deposits, due to rather short transport in a low-energy fluvial system.
